# Bis[2,4-di­bromo-6-(*N*-{4-[(*E*)-1-(benzyl­oxy­imino)­eth­yl]phen­yl}carboximido­yl)phenolato]copper(II)

**DOI:** 10.1107/S1600536813009847

**Published:** 2013-04-13

**Authors:** Xiao-Bing Li, Xiao-Jun Li, Wei-Sheng Meng, Yu-Jie Zhang, Gang Li

**Affiliations:** a Information Centre, Gansu Institute of Forestry Survey and Planning, Lanzhou 730020, People’s Republic of China; b College of Earth and Environmental Scieces, Lanzhou University, Lanzhou 730000, People’s Republic of China; c Zhaosheng Office of Gansu Province, Lanzhou 730030, People’s Republic of China; d School of Chemical and Biological Engineering, Lanzhou Jiaotong University, Lanzhou 730070, People’s Republic of China

## Abstract

In the title complex, [Cu(C_22_H_17_Br_2_N_2_O_2_)_2_], the Cu^II^ ion is four-coordinated in a *trans*-CuN_2_O_2_ square-planar geometry by two phenolate O and two imino N atoms from two deprotonated *N*,*O*-bidentate ligands. In the crystal, the packing of the mol­ecules is controlled by C—H⋯π and π–π inter­actions [centroid–centroid distances = 3.568 (3), 3.678 (2), 3.717 (3) and 3.799 (2) Å] and weak Br⋯Br halogen bonds [3.508 (4) Å], linking the mol­ecules into an infinite three-dimensional supra­molecular network.

## Related literature

For background to oxime-based salen-type tetra­dentate ligands, see: Akine *et al.* (2001 ▶); Dong & Ding (2007 ▶); Dong *et al.* (2012 ▶); Bertolasi *et al.* (1982 ▶); Tarafder *et al.* (2002 ▶). For the synthesis and related crystal structures, see: Zhao & Ng (2010 ▶); Zhao *et al.* (2012 ▶); Dong *et al.* (2010 ▶).
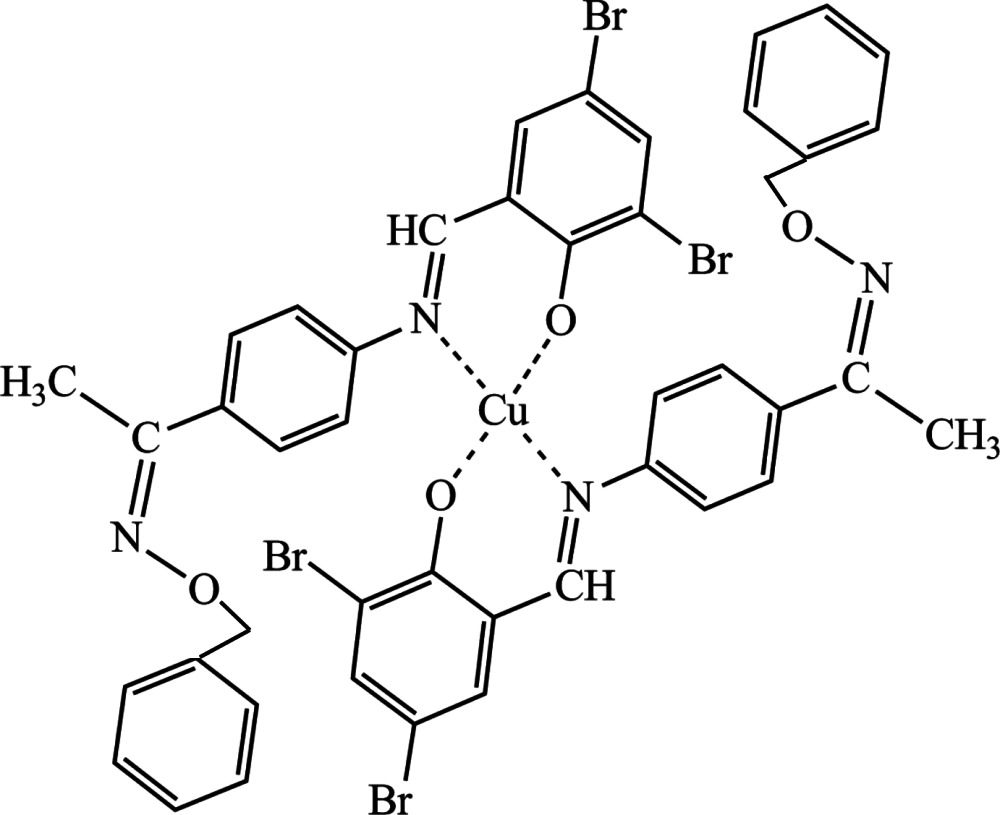


## Experimental

### 

#### Crystal data

[Cu(C_22_H_17_Br_2_N_2_O_2_)_2_]
*M* *_r_* = 1065.93
Monoclinic, 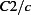

*a* = 27.4484 (7) Å
*b* = 13.3116 (3) Å
*c* = 22.3609 (5) Å
β = 91.165 (2)°
*V* = 8168.6 (3) Å^3^
*Z* = 8
Mo *K*α radiation
μ = 4.50 mm^−1^
*T* = 293 K
0.30 × 0.21 × 0.10 mm

#### Data collection

Bruker SMART 1000 CCD area-detector diffractometer
Absorption correction: multi-scan (*SADABS*; Sheldrick, 1996 ▶) *T*_min_ = 0.346, *T*_max_ = 0.662
27775 measured reflections
7199 independent reflections
4682 reflections with *I* > 2σ(*I*)
*R*_int_ = 0.063

#### Refinement

*R*[*F* ^2^ > 2σ(*F* ^2^)] = 0.041
*wR*(*F* ^2^) = 0.078
*S* = 1.01
7199 reflections
514 parameters
H-atom parameters constrained
Δρ_max_ = 0.38 e Å^−3^
Δρ_min_ = −0.36 e Å^−3^

### 

Data collection: *SMART* (Bruker, 2001 ▶); cell refinement: *SAINT* (Bruker, 2001 ▶); data reduction: *SAINT*; program(s) used to solve structure: *SHELXS97* (Sheldrick, 2008 ▶); program(s) used to refine structure: *SHELXL97* (Sheldrick, 2008 ▶); molecular graphics: *SHELXTL* (Sheldrick, 2008 ▶); software used to prepare material for publication: *SHELXTL*.

## Supplementary Material

Crystal structure: contains datablock(s) global, I. DOI: 10.1107/S1600536813009847/zl2543sup1.cif

Structure factors: contains datablock(s) I. DOI: 10.1107/S1600536813009847/zl2543Isup2.hkl

Additional supplementary materials:  crystallographic information; 3D view; checkCIF report

## Figures and Tables

**Table 1:** Hydrogen-bond geometry (Å, °) *Cg*1 and *Cg*2 are the centroids of the Cu/O4/C33–C31/N4 and C10–C15 rings, respectively.

*D*—H⋯*A*	*D*—H	H⋯*A*	*D*⋯*A*	*D*—H⋯*A*
C19—H19⋯*Cg*1^i^	0.93	2.82	3.575 (5)	118
C16—H16*B*⋯*Cg*2^i^	0.97	2.96	3.511 (5)	117

Symmetry code: (i) 
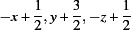
.

**Fig. 1.**
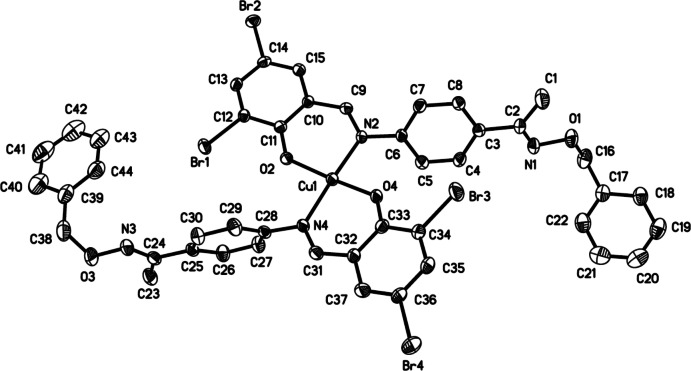
The molecular structure of the title complex with the atom numbering scheme, showing 30% probability displacement ellipsoids. All the H atoms on carbon have been omitted for clarity.

**Fig. 2.**
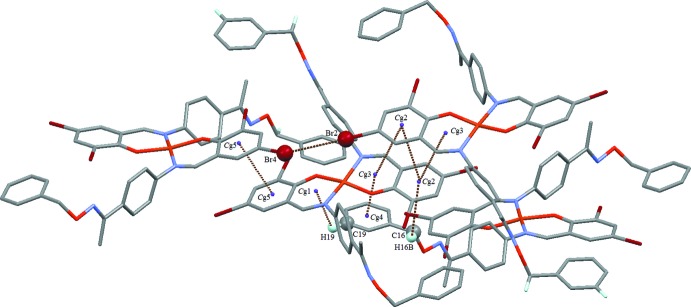
View of the C—H···π and π···π interactions of the title complex (hydrogen atoms, except those forming hydrogen bonds, are omitted for clarity).

**Fig. 3.**
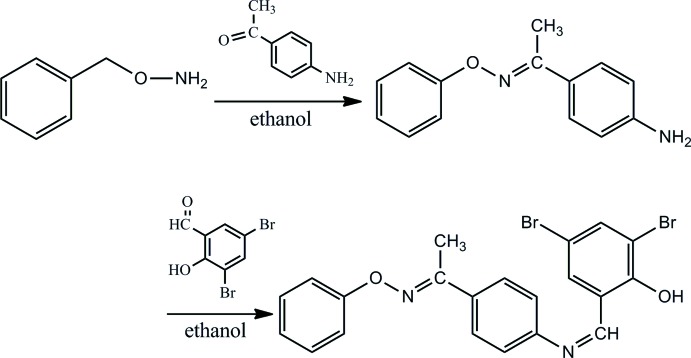
Synthetic route to 1-(4-{[(E)-3,5-dibromo-2-hydroxybenzylidene]amino} phenyl)ethanone O-benzyloxime

## supplementary crystallographic information

### Comment

Cu^II^ complexes with Schiff base ligands have been widely investigated in coordination chemistry and biological chemistry (Akine *et al.*, 2001; Dong *et al.*, 2012). In the last few years there has been a burgeoning effort to identify the biological activities of Cu^II^ ions, primarily through techniques associated with the interface of biology/biochemistry/coordination chemistry (Dong *et al.*, 2007; Tarafder *et al.*, 2002). The oxime moiety can both donate and accept hydrogen bonds, which makes it a very interesting building block in supramolecular chemistry (Bertolasi *et al.*, 1982). Herein a new double functional group ligand bearing both a Schiff base and an oxime group, 2,4-dibromo-6-(*N*-{4-[(*E*)-1-(phenoxyimino)ethyl]phenyl}carboximidoyl)phenol and its Cu^II^ complex, [Cu(C_22_H_17_Br_2_N_2_O_2_)_2_], are reported.

The structure of the title complex is shown in Fig. 1. In the title complex, all bond lengths and angles are in normal ranges. The molecule has approximate chemical inversion symmetry, but no crystallographic inversion symmetry. The Cu^II^ ion is four-coordinated in a *trans*-CuN_2_O_2_ square-planar geometry, with two phenolate O and two imino N atoms from two deprotonated *N*,*O*-bidentate ligands (Dong *et al.*, 2010). The copper atom of [Cu(C_22_H_17_Br_2_N_2_O_2_)_2_] has a square planar geometry distorted tetrahedrally by 13.91 (5)° (defined by the angle between two sets of N—Cu—O planes). A similar distortion has also been observed for the ethoxyl analogue of the title complex (23.33 (3)°) (Zhao *et al.*, 2012).

In the crystal structure, the molecules are linked into an infinite supramolecular network by two C—H···π interactions, C19—H19···*Cg*1 (N4/Cu1/O4/C31—C33) and C16—H16B···*Cg*2 (C10—C15), and four intermolecular π···π stacking interactions, *Cg*2^i^···*Cg*3 (Cu1/O2/N2/C9—C11) [3.799 (2) Å], *Cg*3···*Cg*4^ii^ (C17—C22) [3.568 (3)Å], *Cg*2···*Cg*2^i^ [3.678 (2) Å] and *Cg*5 (C32—C37)···*Cg*5^iii^ [3.717 (3) Å] [symmetry codes: (i) 1/2 - *x*,3/2 - *y*,1 - *z*, (ii) *x*, 1-*y*, -1/2+*z*, (iii) 1-*x*, *y*, 3/2-*z*] (Fig. 2). In addition, the crystal packing is further stabilized by weak intermolecular Br···Br halogen bonds with a distance of 3.508 (4) Å.

### Experimental

1-(4-{[(*E*)-3,5-dibromo-2-hydroxybenzylidene]amino}phenyl)ethanone *O*-benzyloxime was synthesized by reaction of *O*-benzylhydroxylamine, 4-aminoacetophenone and 3,5-dibromosalicylaldehyde (Zhao *et al.*, 2010).

To an ethanol solution (6 ml) of *O*-benzylhydroxylamine (340.9 mg, 3 mmol) was added an ethanol solution (12 ml) of 4-aminoacetophenone (349.5 mg, 3 mmol) and 3 drops of acetic acid. The reaction solution was stirred at 338 K for 24 h. The solvent was evaporated under vacuo. After cooling to room temperature, the formed precipitate was filtered off and washed successively with ethanol and ethanol/water (1:4), respectively, resulting in 640.6 mg of ({4-amino}phenyl)ethanone *O*-benzyloxime as a crystalline solid. Yield, 92.8%, m.p. 348–350 K. Anal. Calcd. for C_15_H_16_N_2O_ (%): C, 74.97; H, 6.71; N, 11.66. Found: C, 74.68; H, 6.80; N, 11.52.

To an ethanol solution (4 ml) of ({4-amino}phenyl)ethanone *O*-benzyloxime (213.2 mg, 0.89 mmol) was added an ethanol solution (2 ml) of 3,5-dibromosalicylaldehyde (2 49.3 mg, 0.89 mmol). The reaction solution was stirred at 333 K for 18 h. After cooling to room temperature, the formed precipitate was filtered off and washed successively with ethanol and ethanol/n-hexane (1:4), respectively. The product was dried *in vacuo* and purified by recrystallization from ethanol to yield 273.3 mg of 1-(4-{[(*E*)-3,5-dibromo-2-hydroxybenzylidene]amino}phenyl)ethanone *O*-benzyloxime. Yield, 59.1%. m.p. 441–442 K. IR: ν C=N, 1608 cm^-1^ and ν Ar—O, 1198 cm^-1^. Anal. Calcd. for C_22_H_18_Br_2_N_2_O_2_ (%): C, 52.62; H, 3.61; N, 5.58. Found: C, 52.49; H, 3.57, N, 5.53.

A pale-blue methanol solution (3 ml) of Cu^II^ acetate monohydrate (0.5 mg, 0.001 mmol) was added dropwise to a red ethyl acetate solution (3 ml) of 1-(4-{[(*E*)-3,5-dibromo-2-hydroxybenzylidene]amino}phenyl)ethanone *O*-benzyloxime (2.6 mg, 0.002 mmol) at room temperature. Upon mixing, the color of the solution turned slowly to brown and the solution was allowed to stand at room temperature for about one week. The solvent was partially evaporated and brown block-like single crystals suitable for X-ray crystallographic analysis were obtained. IR: ν C=N, 1605 cm^-1^ and ν Ar—O, 1186 cm^-1^. Anal. Calcd. for [Cu(C_22_H_17_Br_2_N_2_O_2_)_2_] (C_44_H_34_Br_4_CuN_4_O_4_) (%): C, 49.58; H, 3.22; N, 5.26; Cu, 5.96. Found: C, 49.75; H, 3.13; N, 5.06; Cu, 5.72.

### Refinement

H atoms were placed in calculated positions and non-H atoms were refined anisotropically. H atoms were treated as riding atoms with distances C—H = 0.96 Å (CH_3_) and 0.93 Å (CH). The isotropic displacement parameters for all H atoms were set equal to 1.2 or 1.5 *U*_eq_ of the carrier atom.

### Crystal data

**Table:**

[Cu(C_22_H_17_Br_2_N_2_O_2_)_2_]	*F*(000) = 4216
*M**_r_* = 1065.93	*D*_x_ = 1.733 Mg m^−^^3^
Monoclinic, *C*2/*c*	Mo *K*α radiation, λ = 0.71073 Å
Hall symbol: -C 2yc	Cell parameters from 5329 reflections
*a* = 27.4484 (7) Å	θ = 2.5–28.4°
*b* = 13.3116 (3) Å	µ = 4.50 mm^−^^1^
*c* = 22.3609 (5) Å	*T* = 293 K
β = 91.165 (2)°	Block-like, brown
*V* = 8168.6 (3) Å^3^	0.30 × 0.21 × 0.10 mm
*Z* = 8	

### Data collection

**Table:**

Bruker SMART 1000 CCD area-detector diffractometer	7199 independent reflections
Radiation source: fine-focus sealed tube	4682 reflections with *I* > 2σ(*I*)
Graphite monochromator	*R*_int_ = 0.063
phi and ω scans	θ_max_ = 25.0°, θ_min_ = 2.5°
Absorption correction: multi-scan (*SADABS*; Sheldrick, 1996)	*h* = −32→32
*T*_min_ = 0.346, *T*_max_ = 0.662	*k* = −15→15
27775 measured reflections	*l* = −26→26

### Refinement

**Table:**

Refinement on *F*^2^	Primary atom site location: structure-invariant direct methods
Least-squares matrix: full	Secondary atom site location: difference Fourier map
*R*[*F*^2^ > 2σ(*F*^2^)] = 0.041	Hydrogen site location: inferred from neighbouring sites
*wR*(*F*^2^) = 0.078	H-atom parameters constrained
*S* = 1.01	*w* = 1/[σ^2^(*F*_o_^2^) + (0.0217*P*)^2^ + 4.428*P*] where *P* = (*F*_o_^2^ + 2*F*_c_^2^)/3
7199 reflections	(Δ/σ)_max_ = 0.001
514 parameters	Δρ_max_ = 0.38 e Å^−^^3^
0 restraints	Δρ_min_ = −0.36 e Å^−^^3^

### Special details

**Table:**

Geometry. All e.s.d.'s (except the e.s.d. in the dihedral angle between two l.s. planes) are estimated using the full covariance matrix. The cell e.s.d.'s are taken into account individually in the estimation of e.s.d.'s in distances, angles and torsion angles; correlations between e.s.d.'s in cell parameters are only used when they are defined by crystal symmetry. An approximate (isotropic) treatment of cell e.s.d.'s is used for estimating e.s.d.'s involving l.s. planes.
Refinement. Refinement of *F*^2^ against ALL reflections. The weighted *R*-factor *wR* and goodness of fit *S* are based on *F*^2^, conventional *R*-factors *R* are based on *F*, with *F* set to zero for negative *F*^2^. The threshold expression of *F*^2^ > σ(*F*^2^) is used only for calculating *R*-factors(gt) *etc*. and is not relevant to the choice of reflections for refinement. *R*-factors based on *F*^2^ are statistically about twice as large as those based on *F*, and *R*- factors based on ALL data will be even larger.

### Fractional atomic coordinates and isotropic or equivalent isotropic displacement parameters (Å^2^)

**Table:**

	*x*	*y*	*z*	*U*_iso_*/*U*_eq_	
Cu1	0.393085 (19)	0.80478 (4)	0.56844 (2)	0.03676 (15)	
Br1	0.313417 (19)	1.00341 (3)	0.41540 (2)	0.05451 (16)	
Br2	0.202079 (18)	0.66785 (3)	0.34341 (2)	0.04794 (14)	
Br3	0.51425 (2)	0.59414 (4)	0.66431 (3)	0.07055 (19)	
Br4	0.61488 (2)	0.93975 (4)	0.74609 (3)	0.0720 (2)	
N1	0.40018 (14)	0.3244 (3)	0.75569 (17)	0.0462 (10)	
N2	0.36392 (12)	0.6673 (2)	0.56013 (14)	0.0334 (9)	
N3	0.34560 (16)	1.3351 (3)	0.44525 (19)	0.0558 (12)	
N4	0.41632 (12)	0.9450 (2)	0.58442 (15)	0.0369 (9)	
O1	0.41180 (12)	0.2328 (2)	0.78465 (14)	0.0582 (9)	
O2	0.35153 (10)	0.85865 (18)	0.50786 (12)	0.0377 (7)	
O3	0.32379 (14)	1.4261 (2)	0.42526 (16)	0.0681 (11)	
O4	0.44441 (10)	0.74996 (19)	0.61628 (12)	0.0427 (8)	
C1	0.4484 (2)	0.2458 (4)	0.6779 (2)	0.0712 (17)	
H1A	0.4798	0.2719	0.6680	0.107*	
H1B	0.4326	0.2197	0.6425	0.107*	
H1C	0.4523	0.1929	0.7068	0.107*	
C2	0.41821 (17)	0.3274 (3)	0.7033 (2)	0.0403 (11)	
C3	0.40625 (15)	0.4177 (3)	0.66696 (19)	0.0356 (11)	
C4	0.38720 (17)	0.5041 (3)	0.69259 (19)	0.0466 (12)	
H4	0.3828	0.5063	0.7337	0.056*	
C5	0.37466 (17)	0.5867 (3)	0.6582 (2)	0.0457 (12)	
H5	0.3629	0.6444	0.6765	0.055*	
C6	0.37943 (15)	0.5841 (3)	0.59716 (19)	0.0352 (11)	
C7	0.39876 (15)	0.4995 (3)	0.57136 (19)	0.0406 (11)	
H7	0.4030	0.4976	0.5302	0.049*	
C8	0.41192 (16)	0.4171 (3)	0.60602 (19)	0.0390 (11)	
H8	0.4248	0.3605	0.5877	0.047*	
C9	0.33200 (15)	0.6446 (3)	0.51906 (17)	0.0354 (11)	
H9	0.3225	0.5776	0.5175	0.042*	
C10	0.30944 (15)	0.7097 (3)	0.47563 (17)	0.0312 (10)	
C11	0.32100 (15)	0.8134 (3)	0.47231 (18)	0.0331 (10)	
C12	0.29653 (15)	0.8671 (3)	0.42596 (18)	0.0325 (10)	
C13	0.26243 (15)	0.8249 (3)	0.38851 (18)	0.0363 (11)	
H13	0.2468	0.8636	0.3593	0.044*	
C14	0.25130 (15)	0.7241 (3)	0.39428 (18)	0.0360 (11)	
C15	0.27448 (15)	0.6672 (3)	0.43670 (18)	0.0372 (11)	
H15	0.2670	0.5992	0.4399	0.045*	
C16	0.38905 (19)	0.2287 (4)	0.8420 (2)	0.0617 (15)	
H16A	0.3783	0.1605	0.8494	0.074*	
H16B	0.3605	0.2717	0.8413	0.074*	
C17	0.42285 (17)	0.2612 (4)	0.8916 (2)	0.0478 (13)	
C18	0.44426 (19)	0.1915 (4)	0.9290 (2)	0.0588 (14)	
H18	0.4375	0.1237	0.9235	0.071*	
C19	0.4758 (2)	0.2208 (5)	0.9748 (2)	0.0738 (17)	
H19	0.4898	0.1729	1.0000	0.089*	
C20	0.4861 (2)	0.3197 (6)	0.9829 (3)	0.0772 (18)	
H20	0.5074	0.3395	1.0135	0.093*	
C21	0.4656 (2)	0.3894 (5)	0.9463 (3)	0.0780 (18)	
H21	0.4727	0.4571	0.9519	0.094*	
C22	0.4340 (2)	0.3602 (4)	0.9009 (2)	0.0674 (16)	
H22	0.4199	0.4087	0.8761	0.081*	
C23	0.28957 (18)	1.3574 (3)	0.5297 (2)	0.0584 (14)	
H23A	0.3038	1.3891	0.5644	0.088*	
H23B	0.2659	1.3089	0.5419	0.088*	
H23C	0.2740	1.4073	0.5049	0.088*	
C24	0.32845 (18)	1.3061 (3)	0.4953 (2)	0.0445 (12)	
C25	0.35100 (17)	1.2114 (3)	0.5185 (2)	0.0408 (12)	
C26	0.33122 (18)	1.1602 (3)	0.5662 (2)	0.0509 (13)	
H26	0.3039	1.1860	0.5848	0.061*	
C27	0.35166 (17)	1.0705 (3)	0.5865 (2)	0.0494 (13)	
H27	0.3378	1.0361	0.6181	0.059*	
C28	0.39222 (16)	1.0333 (3)	0.5599 (2)	0.0364 (11)	
C29	0.41171 (17)	1.0817 (3)	0.5122 (2)	0.0496 (13)	
H29	0.4387	1.0548	0.4935	0.060*	
C30	0.39136 (18)	1.1708 (3)	0.4915 (2)	0.0502 (13)	
H30	0.4050	1.2036	0.4592	0.060*	
C31	0.45492 (16)	0.9665 (3)	0.61517 (19)	0.0432 (12)	
H31	0.4622	1.0344	0.6191	0.052*	
C32	0.48802 (16)	0.8982 (3)	0.64407 (19)	0.0412 (12)	
C33	0.48142 (17)	0.7931 (3)	0.64184 (18)	0.0398 (11)	
C34	0.51902 (17)	0.7353 (3)	0.66960 (19)	0.0432 (12)	
C35	0.55761 (17)	0.7769 (3)	0.70001 (19)	0.0468 (13)	
H35	0.5808	0.7359	0.7186	0.056*	
C36	0.56205 (17)	0.8805 (3)	0.7030 (2)	0.0482 (13)	
C37	0.52853 (17)	0.9402 (3)	0.6752 (2)	0.0504 (13)	
H37	0.5323	1.0096	0.6765	0.060*	
C38	0.3297 (2)	1.4292 (4)	0.3611 (2)	0.0734 (17)	
H38A	0.3619	1.4047	0.3515	0.088*	
H38B	0.3271	1.4982	0.3474	0.088*	
C39	0.2922 (2)	1.3669 (4)	0.3295 (2)	0.0670 (16)	
C40	0.2531 (3)	1.4106 (5)	0.2979 (3)	0.094 (2)	
H40	0.2512	1.4800	0.2937	0.112*	
C41	0.2170 (3)	1.3490 (7)	0.2726 (3)	0.099 (2)	
H41	0.1913	1.3782	0.2512	0.119*	
C42	0.2187 (3)	1.2477 (7)	0.2784 (3)	0.100 (2)	
H42	0.1936	1.2082	0.2625	0.120*	
C43	0.2571 (3)	1.2046 (5)	0.3074 (2)	0.088 (2)	
H43	0.2589	1.1350	0.3104	0.106*	
C44	0.2934 (2)	1.2630 (4)	0.3324 (2)	0.0703 (17)	
H44	0.3196	1.2319	0.3518	0.084*	

### Atomic displacement parameters (Å^2^)

**Table:**

	*U*^11^	*U*^22^	*U*^33^	*U*^12^	*U*^13^	*U*^23^
Cu1	0.0360 (3)	0.0314 (3)	0.0423 (3)	0.0009 (2)	−0.0114 (3)	0.0010 (2)
Br1	0.0655 (4)	0.0335 (2)	0.0637 (3)	−0.0007 (2)	−0.0166 (3)	0.0084 (2)
Br2	0.0466 (3)	0.0513 (3)	0.0453 (3)	−0.0020 (2)	−0.0140 (2)	−0.0079 (2)
Br3	0.0702 (4)	0.0431 (3)	0.0970 (4)	0.0108 (3)	−0.0295 (4)	0.0048 (3)
Br4	0.0546 (4)	0.0807 (4)	0.0791 (4)	−0.0073 (3)	−0.0361 (3)	−0.0002 (3)
N1	0.048 (3)	0.042 (2)	0.049 (3)	0.0007 (19)	−0.001 (2)	0.015 (2)
N2	0.036 (2)	0.0293 (18)	0.035 (2)	0.0047 (17)	−0.0067 (19)	0.0026 (16)
N3	0.069 (3)	0.037 (2)	0.061 (3)	0.010 (2)	−0.012 (3)	0.007 (2)
N4	0.034 (2)	0.0289 (19)	0.047 (2)	0.0040 (17)	−0.013 (2)	−0.0001 (17)
O1	0.067 (2)	0.055 (2)	0.053 (2)	0.0056 (18)	0.0068 (19)	0.0220 (17)
O2	0.0375 (18)	0.0325 (15)	0.0427 (18)	−0.0023 (14)	−0.0125 (16)	0.0009 (14)
O3	0.095 (3)	0.043 (2)	0.065 (2)	0.018 (2)	−0.006 (2)	0.0105 (18)
O4	0.044 (2)	0.0315 (16)	0.0516 (19)	0.0005 (14)	−0.0208 (17)	0.0043 (14)
C1	0.085 (4)	0.065 (4)	0.063 (4)	0.029 (3)	0.012 (3)	0.016 (3)
C2	0.040 (3)	0.039 (3)	0.042 (3)	0.005 (2)	−0.003 (2)	0.008 (2)
C3	0.035 (3)	0.035 (2)	0.037 (3)	0.000 (2)	0.001 (2)	0.002 (2)
C4	0.061 (3)	0.048 (3)	0.031 (3)	0.006 (3)	0.002 (2)	0.005 (2)
C5	0.057 (3)	0.038 (3)	0.042 (3)	0.008 (2)	0.000 (3)	−0.002 (2)
C6	0.035 (3)	0.031 (2)	0.039 (3)	0.002 (2)	−0.010 (2)	0.003 (2)
C7	0.046 (3)	0.045 (3)	0.031 (3)	0.002 (2)	0.001 (2)	0.003 (2)
C8	0.042 (3)	0.035 (2)	0.039 (3)	0.004 (2)	0.001 (2)	0.001 (2)
C9	0.037 (3)	0.033 (2)	0.036 (3)	−0.002 (2)	−0.003 (2)	−0.003 (2)
C10	0.029 (3)	0.034 (2)	0.030 (2)	0.002 (2)	0.000 (2)	0.001 (2)
C11	0.031 (3)	0.034 (2)	0.034 (3)	0.004 (2)	0.001 (2)	−0.002 (2)
C12	0.030 (3)	0.033 (2)	0.034 (2)	0.004 (2)	0.001 (2)	0.002 (2)
C13	0.038 (3)	0.040 (3)	0.031 (2)	0.007 (2)	−0.002 (2)	0.002 (2)
C14	0.034 (3)	0.043 (3)	0.031 (2)	0.001 (2)	−0.005 (2)	−0.007 (2)
C15	0.039 (3)	0.034 (2)	0.038 (3)	−0.004 (2)	−0.001 (2)	0.001 (2)
C16	0.055 (4)	0.073 (4)	0.057 (3)	−0.007 (3)	0.007 (3)	0.028 (3)
C17	0.039 (3)	0.058 (3)	0.047 (3)	0.000 (3)	0.012 (3)	0.012 (3)
C18	0.060 (4)	0.062 (3)	0.055 (3)	0.009 (3)	0.012 (3)	0.013 (3)
C19	0.065 (4)	0.100 (5)	0.058 (4)	0.014 (4)	0.007 (3)	0.020 (4)
C20	0.060 (4)	0.115 (6)	0.057 (4)	−0.015 (4)	0.009 (3)	−0.001 (4)
C21	0.082 (5)	0.077 (4)	0.076 (4)	−0.013 (4)	0.020 (4)	−0.006 (4)
C22	0.065 (4)	0.067 (4)	0.070 (4)	0.004 (3)	0.011 (3)	0.017 (3)
C23	0.055 (3)	0.042 (3)	0.078 (4)	0.006 (3)	−0.006 (3)	0.007 (3)
C24	0.045 (3)	0.033 (3)	0.055 (3)	0.001 (2)	−0.009 (3)	−0.002 (2)
C25	0.040 (3)	0.030 (2)	0.051 (3)	−0.003 (2)	−0.010 (3)	0.001 (2)
C26	0.047 (3)	0.047 (3)	0.058 (3)	0.015 (3)	0.001 (3)	0.007 (3)
C27	0.050 (3)	0.043 (3)	0.056 (3)	0.009 (3)	0.004 (3)	0.009 (2)
C28	0.033 (3)	0.029 (2)	0.048 (3)	−0.003 (2)	−0.010 (2)	0.000 (2)
C29	0.040 (3)	0.044 (3)	0.065 (3)	0.008 (2)	0.004 (3)	0.006 (3)
C30	0.051 (3)	0.043 (3)	0.057 (3)	0.002 (3)	0.003 (3)	0.012 (2)
C31	0.043 (3)	0.034 (2)	0.052 (3)	0.002 (2)	−0.008 (3)	−0.002 (2)
C32	0.040 (3)	0.036 (3)	0.047 (3)	0.002 (2)	−0.013 (2)	0.003 (2)
C33	0.039 (3)	0.044 (3)	0.036 (3)	0.005 (2)	−0.004 (2)	0.000 (2)
C34	0.044 (3)	0.042 (3)	0.043 (3)	0.006 (2)	−0.007 (3)	0.002 (2)
C35	0.041 (3)	0.054 (3)	0.045 (3)	0.007 (3)	−0.010 (3)	0.006 (2)
C36	0.040 (3)	0.056 (3)	0.049 (3)	0.000 (2)	−0.018 (3)	0.001 (2)
C37	0.049 (3)	0.043 (3)	0.058 (3)	−0.003 (2)	−0.016 (3)	−0.004 (2)
C38	0.098 (5)	0.059 (4)	0.063 (4)	0.017 (3)	0.012 (4)	0.020 (3)
C39	0.077 (5)	0.078 (4)	0.046 (3)	0.030 (4)	0.003 (3)	0.007 (3)
C40	0.111 (6)	0.103 (5)	0.067 (4)	0.053 (5)	0.002 (4)	−0.005 (4)
C41	0.085 (6)	0.144 (7)	0.069 (5)	0.057 (5)	−0.013 (4)	−0.006 (5)
C42	0.079 (5)	0.150 (7)	0.071 (5)	0.015 (5)	−0.003 (4)	−0.008 (5)
C43	0.096 (5)	0.108 (5)	0.061 (4)	0.003 (5)	−0.012 (4)	0.003 (4)
C44	0.073 (4)	0.081 (4)	0.056 (4)	0.010 (4)	−0.011 (3)	0.003 (3)

### Geometric parameters (Å, º)

**Table:**

Cu1—O2	1.894 (2)	C17—C18	1.373 (6)
Cu1—O4	1.897 (3)	C18—C19	1.382 (7)
Cu1—N4	2.002 (3)	C18—H18	0.9300
Cu1—N2	2.005 (3)	C19—C20	1.358 (7)
Br1—C12	1.888 (4)	C19—H19	0.9300
Br2—C14	1.902 (4)	C20—C21	1.352 (7)
Br3—C34	1.887 (4)	C20—H20	0.9300
Br4—C36	1.896 (4)	C21—C22	1.377 (7)
N1—C2	1.282 (5)	C21—H21	0.9300
N1—O1	1.413 (4)	C22—H22	0.9300
N2—C9	1.292 (4)	C23—C24	1.494 (6)
N2—C6	1.442 (5)	C23—H23A	0.9600
N3—C24	1.283 (6)	C23—H23B	0.9600
N3—O3	1.420 (4)	C23—H23C	0.9600
N4—C31	1.284 (5)	C24—C25	1.492 (6)
N4—C28	1.451 (5)	C25—C30	1.383 (6)
O1—C16	1.438 (5)	C25—C26	1.385 (6)
O2—C11	1.292 (4)	C26—C27	1.392 (5)
O3—C38	1.447 (6)	C26—H26	0.9300
O4—C33	1.290 (5)	C27—C28	1.366 (6)
C1—C2	1.487 (6)	C27—H27	0.9300
C1—H1A	0.9600	C28—C29	1.364 (6)
C1—H1B	0.9600	C29—C30	1.387 (6)
C1—H1C	0.9600	C29—H29	0.9300
C2—C3	1.483 (5)	C30—H30	0.9300
C3—C8	1.375 (5)	C31—C32	1.430 (5)
C3—C4	1.393 (5)	C31—H31	0.9300
C4—C5	1.381 (5)	C32—C33	1.411 (5)
C4—H4	0.9300	C32—C37	1.415 (5)
C5—C6	1.375 (6)	C33—C34	1.420 (5)
C5—H5	0.9300	C34—C35	1.364 (6)
C6—C7	1.377 (5)	C35—C36	1.386 (6)
C7—C8	1.387 (5)	C35—H35	0.9300
C7—H7	0.9300	C36—C37	1.357 (6)
C8—H8	0.9300	C37—H37	0.9300
C9—C10	1.433 (5)	C38—C39	1.490 (7)
C9—H9	0.9300	C38—H38A	0.9700
C10—C15	1.402 (5)	C38—H38B	0.9700
C10—C11	1.419 (5)	C39—C44	1.385 (7)
C11—C12	1.417 (5)	C39—C40	1.398 (7)
C12—C13	1.365 (5)	C40—C41	1.396 (9)
C13—C14	1.382 (5)	C40—H40	0.9300
C13—H13	0.9300	C41—C42	1.355 (9)
C14—C15	1.362 (5)	C41—H41	0.9300
C15—H15	0.9300	C42—C43	1.354 (8)
C16—C17	1.496 (6)	C42—H42	0.9300
C16—H16A	0.9700	C43—C44	1.375 (7)
C16—H16B	0.9700	C43—H43	0.9300
C17—C22	1.368 (6)	C44—H44	0.9300
			
O2—Cu1—O4	168.03 (13)	C21—C20—C19	120.0 (6)
O2—Cu1—N4	87.69 (12)	C21—C20—H20	120.0
O4—Cu1—N4	91.59 (12)	C19—C20—H20	120.0
O2—Cu1—N2	92.63 (12)	C20—C21—C22	120.0 (6)
O4—Cu1—N2	89.52 (12)	C20—C21—H21	120.0
N4—Cu1—N2	173.08 (15)	C22—C21—H21	120.0
C2—N1—O1	111.0 (4)	C17—C22—C21	121.3 (5)
C9—N2—C6	114.6 (3)	C17—C22—H22	119.3
C9—N2—Cu1	122.9 (3)	C21—C22—H22	119.3
C6—N2—Cu1	122.4 (2)	C24—C23—H23A	109.5
C24—N3—O3	111.8 (4)	C24—C23—H23B	109.5
C31—N4—C28	112.7 (3)	H23A—C23—H23B	109.5
C31—N4—Cu1	124.0 (3)	C24—C23—H23C	109.5
C28—N4—Cu1	123.2 (2)	H23A—C23—H23C	109.5
N1—O1—C16	110.1 (3)	H23B—C23—H23C	109.5
C11—O2—Cu1	129.6 (2)	N3—C24—C25	113.6 (5)
N3—O3—C38	106.3 (4)	N3—C24—C23	126.2 (4)
C33—O4—Cu1	130.3 (3)	C25—C24—C23	120.2 (4)
C2—C1—H1A	109.5	C30—C25—C26	118.3 (4)
C2—C1—H1B	109.5	C30—C25—C24	120.6 (4)
H1A—C1—H1B	109.5	C26—C25—C24	121.1 (5)
C2—C1—H1C	109.5	C25—C26—C27	120.8 (5)
H1A—C1—H1C	109.5	C25—C26—H26	119.6
H1B—C1—H1C	109.5	C27—C26—H26	119.6
N1—C2—C3	116.2 (4)	C28—C27—C26	119.7 (5)
N1—C2—C1	123.7 (4)	C28—C27—H27	120.2
C3—C2—C1	120.1 (4)	C26—C27—H27	120.2
C8—C3—C4	117.6 (4)	C29—C28—C27	120.4 (4)
C8—C3—C2	120.7 (4)	C29—C28—N4	119.6 (4)
C4—C3—C2	121.6 (4)	C27—C28—N4	119.9 (4)
C5—C4—C3	121.4 (4)	C28—C29—C30	120.2 (5)
C5—C4—H4	119.3	C28—C29—H29	119.9
C3—C4—H4	119.3	C30—C29—H29	119.9
C6—C5—C4	120.3 (4)	C25—C30—C29	120.6 (5)
C6—C5—H5	119.8	C25—C30—H30	119.7
C4—C5—H5	119.8	C29—C30—H30	119.7
C5—C6—C7	118.8 (4)	N4—C31—C32	127.6 (4)
C5—C6—N2	121.2 (4)	N4—C31—H31	116.2
C7—C6—N2	120.0 (4)	C32—C31—H31	116.2
C6—C7—C8	120.7 (4)	C33—C32—C37	120.5 (4)
C6—C7—H7	119.6	C33—C32—C31	122.3 (4)
C8—C7—H7	119.6	C37—C32—C31	117.2 (4)
C3—C8—C7	121.1 (4)	O4—C33—C32	123.8 (4)
C3—C8—H8	119.5	O4—C33—C34	120.7 (4)
C7—C8—H8	119.5	C32—C33—C34	115.5 (4)
N2—C9—C10	128.1 (4)	C35—C34—C33	123.2 (4)
N2—C9—H9	115.9	C35—C34—Br3	119.2 (3)
C10—C9—H9	115.9	C33—C34—Br3	117.6 (3)
C15—C10—C11	120.8 (4)	C34—C35—C36	119.6 (4)
C15—C10—C9	117.2 (4)	C34—C35—H35	120.2
C11—C10—C9	122.0 (4)	C36—C35—H35	120.2
O2—C11—C12	120.4 (4)	C37—C36—C35	120.1 (4)
O2—C11—C10	124.3 (3)	C37—C36—Br4	119.6 (3)
C12—C11—C10	115.2 (4)	C35—C36—Br4	120.3 (3)
C13—C12—C11	123.3 (4)	C36—C37—C32	120.9 (4)
C13—C12—Br1	119.1 (3)	C36—C37—H37	119.6
C11—C12—Br1	117.5 (3)	C32—C37—H37	119.6
C12—C13—C14	119.5 (4)	O3—C38—C39	111.4 (5)
C12—C13—H13	120.2	O3—C38—H38A	109.4
C14—C13—H13	120.2	C39—C38—H38A	109.4
C15—C14—C13	120.3 (4)	O3—C38—H38B	109.4
C15—C14—Br2	120.9 (3)	C39—C38—H38B	109.4
C13—C14—Br2	118.8 (3)	H38A—C38—H38B	108.0
C14—C15—C10	120.8 (4)	C44—C39—C40	117.2 (6)
C14—C15—H15	119.6	C44—C39—C38	121.2 (5)
C10—C15—H15	119.6	C40—C39—C38	121.6 (6)
O1—C16—C17	112.1 (4)	C41—C40—C39	119.4 (6)
O1—C16—H16A	109.2	C41—C40—H40	120.3
C17—C16—H16A	109.2	C39—C40—H40	120.3
O1—C16—H16B	109.2	C42—C41—C40	121.5 (6)
C17—C16—H16B	109.2	C42—C41—H41	119.2
H16A—C16—H16B	107.9	C40—C41—H41	119.2
C22—C17—C18	117.8 (5)	C43—C42—C41	119.4 (7)
C22—C17—C16	121.7 (5)	C43—C42—H42	120.3
C18—C17—C16	120.6 (5)	C41—C42—H42	120.3
C17—C18—C19	121.0 (5)	C42—C43—C44	120.4 (7)
C17—C18—H18	119.5	C42—C43—H43	119.8
C19—C18—H18	119.5	C44—C43—H43	119.8
C20—C19—C18	119.9 (5)	C43—C44—C39	122.0 (6)
C20—C19—H19	120.1	C43—C44—H44	119.0
C18—C19—H19	120.1	C39—C44—H44	119.0
			
O2—Cu1—N2—C9	5.8 (3)	C22—C17—C18—C19	0.4 (8)
O4—Cu1—N2—C9	−162.4 (3)	C16—C17—C18—C19	179.6 (5)
O2—Cu1—N2—C6	−178.4 (3)	C17—C18—C19—C20	−0.5 (8)
O4—Cu1—N2—C6	13.3 (3)	C18—C19—C20—C21	0.3 (9)
O2—Cu1—N4—C31	−165.0 (4)	C19—C20—C21—C22	0.1 (9)
O4—Cu1—N4—C31	3.0 (4)	C18—C17—C22—C21	0.0 (8)
O2—Cu1—N4—C28	12.1 (3)	C16—C17—C22—C21	−179.3 (5)
O4—Cu1—N4—C28	−179.9 (4)	C20—C21—C22—C17	−0.2 (9)
C2—N1—O1—C16	−176.9 (4)	O3—N3—C24—C25	−179.0 (3)
O4—Cu1—O2—C11	93.3 (6)	O3—N3—C24—C23	1.3 (6)
N4—Cu1—O2—C11	−179.9 (4)	N3—C24—C25—C30	9.1 (6)
N2—Cu1—O2—C11	−6.9 (4)	C23—C24—C25—C30	−171.1 (4)
C24—N3—O3—C38	−156.9 (4)	N3—C24—C25—C26	−168.6 (4)
O2—Cu1—O4—C33	79.9 (7)	C23—C24—C25—C26	11.1 (6)
N4—Cu1—O4—C33	−6.5 (4)	C30—C25—C26—C27	0.5 (7)
N2—Cu1—O4—C33	−179.7 (4)	C24—C25—C26—C27	178.2 (4)
O1—N1—C2—C3	176.8 (3)	C25—C26—C27—C28	1.1 (7)
O1—N1—C2—C1	−1.5 (6)	C26—C27—C28—C29	−2.3 (6)
N1—C2—C3—C8	−162.1 (4)	C26—C27—C28—N4	174.3 (4)
C1—C2—C3—C8	16.3 (7)	C31—N4—C28—C29	74.8 (5)
N1—C2—C3—C4	15.4 (7)	Cu1—N4—C28—C29	−102.5 (4)
C1—C2—C3—C4	−166.3 (4)	C31—N4—C28—C27	−101.9 (5)
C8—C3—C4—C5	−0.4 (7)	Cu1—N4—C28—C27	80.7 (5)
C2—C3—C4—C5	−177.9 (4)	C27—C28—C29—C30	2.0 (7)
C3—C4—C5—C6	2.0 (7)	N4—C28—C29—C30	−174.6 (4)
C4—C5—C6—C7	−2.7 (7)	C26—C25—C30—C29	−0.8 (7)
C4—C5—C6—N2	176.4 (4)	C24—C25—C30—C29	−178.5 (4)
C9—N2—C6—C5	−122.6 (4)	C28—C29—C30—C25	−0.5 (7)
Cu1—N2—C6—C5	61.4 (5)	C28—N4—C31—C32	−178.1 (4)
C9—N2—C6—C7	56.4 (5)	Cu1—N4—C31—C32	−0.8 (7)
Cu1—N2—C6—C7	−119.6 (4)	N4—C31—C32—C33	−0.1 (8)
C5—C6—C7—C8	1.8 (7)	N4—C31—C32—C37	179.5 (5)
N2—C6—C7—C8	−177.2 (4)	Cu1—O4—C33—C32	7.5 (7)
C4—C3—C8—C7	−0.5 (7)	Cu1—O4—C33—C34	−172.0 (3)
C2—C3—C8—C7	177.1 (4)	C37—C32—C33—O4	177.3 (4)
C6—C7—C8—C3	−0.3 (7)	C31—C32—C33—O4	−3.2 (7)
C6—N2—C9—C10	179.8 (4)	C37—C32—C33—C34	−3.3 (7)
Cu1—N2—C9—C10	−4.2 (6)	C31—C32—C33—C34	176.2 (4)
N2—C9—C10—C15	−178.1 (4)	O4—C33—C34—C35	−176.4 (4)
N2—C9—C10—C11	0.9 (7)	C32—C33—C34—C35	4.2 (7)
Cu1—O2—C11—C12	−174.0 (3)	O4—C33—C34—Br3	2.9 (6)
Cu1—O2—C11—C10	5.7 (6)	C32—C33—C34—Br3	−176.5 (3)
C15—C10—C11—O2	177.6 (4)	C33—C34—C35—C36	−2.1 (7)
C9—C10—C11—O2	−1.3 (7)	Br3—C34—C35—C36	178.6 (4)
C15—C10—C11—C12	−2.7 (6)	C34—C35—C36—C37	−0.9 (8)
C9—C10—C11—C12	178.4 (4)	C34—C35—C36—Br4	179.0 (4)
O2—C11—C12—C13	−177.2 (4)	C35—C36—C37—C32	1.7 (8)
C10—C11—C12—C13	3.0 (6)	Br4—C36—C37—C32	−178.3 (4)
O2—C11—C12—Br1	4.0 (5)	C33—C32—C37—C36	0.5 (7)
C10—C11—C12—Br1	−175.8 (3)	C31—C32—C37—C36	−179.0 (5)
C11—C12—C13—C14	−1.5 (7)	N3—O3—C38—C39	80.7 (5)
Br1—C12—C13—C14	177.3 (3)	O3—C38—C39—C44	−71.0 (7)
C12—C13—C14—C15	−0.6 (7)	O3—C38—C39—C40	106.2 (6)
C12—C13—C14—Br2	177.9 (3)	C44—C39—C40—C41	1.8 (9)
C13—C14—C15—C10	0.8 (7)	C38—C39—C40—C41	−175.5 (6)
Br2—C14—C15—C10	−177.6 (3)	C39—C40—C41—C42	0.6 (10)
C11—C10—C15—C14	0.9 (6)	C40—C41—C42—C43	−2.5 (11)
C9—C10—C15—C14	179.8 (4)	C41—C42—C43—C44	2.0 (10)
N1—O1—C16—C17	−96.1 (4)	C42—C43—C44—C39	0.5 (10)
O1—C16—C17—C22	75.4 (6)	C40—C39—C44—C43	−2.4 (9)
O1—C16—C17—C18	−103.8 (5)	C38—C39—C44—C43	174.9 (5)

### Hydrogen-bond geometry (Å, º)

Cg1 and Cg2 are the centroids of the Cu/O4/C33–C31/N4 and C10–C15 rings, respectively.

**Table:**

*D*—H···*A*	*D*—H	H···*A*	*D*···*A*	*D*—H···*A*
C19—H19···*Cg*1^i^	0.93	2.82	3.575 (5)	118
C16—H16*B*···*Cg*2^i^	0.97	2.96	3.511 (5)	117

Symmetry code: (i) −*x*+1/2, *y*+3/2, −*z*+1/2.
